# Early and late complications of bariatric operation

**DOI:** 10.1136/tsaco-2018-000219

**Published:** 2018-10-09

**Authors:** Robert Lim, Alec Beekley, Dirk C Johnson, Kimberly A Davis

**Affiliations:** 1 Department of Surgery, Tripler Army Medical Center, Tripler, Honolulu, Hawaii, USA; 2 Department of Surgery, Sidney Kimmel Medical College at Thomas Jefferson University, Philadelphia, Pennsylvania, USA; 3 Department of Surgery, Yale School of Medicine, New Haven, Connecticut, USA

**Keywords:** complications, morbid obesity, acute care surgery

## Abstract

Weight loss surgery is one of the fastest growing segments of the surgical discipline. As with all medical procedures, postoperative complications will occur. Acute care surgeons need to be familiar with the common problems and their management. Although general surgical principles generally apply, diagnoses specific to the various bariatric operations must be considered. There are anatomic considerations which alter management priorities and options for these patients in many instances. These problems present both early or late in the postoperative course. Bariatric operations, in many instances, result in permanent alteration of a patient’s anatomy, which can lead to complications at any time during the course of a patient’s life. Acute care surgeons diagnosing surgical emergencies in postbariatric operation patients must be familiar with the type of surgery performed, as well as the common postbariatric surgical emergencies. In addition, surgeons must not overlook the common causes of an acute surgical abdomen—acute appendicitis, acute diverticulitis, acute pancreatitis, and gallstone disease—for these are still among the most common etiologies of abdominal pathology in these patients.

## Introduction

Weight loss surgery is one of the fastest growing segments of the surgical discipline. As with all medical procedures, postoperative complications will occur. Acute care surgeons need to be familiar with the common problems and their management. Although general surgical principles generally apply, diagnoses specific to the various bariatric operations must be considered. There may be anatomic considerations which alter management priorities and options for these patients in many instances. These problems present both early or late in the postoperative course.

Bariatric operations result in permanent alteration of a patient’s anatomy, which can lead to complications at any time during the course of a patient’s life. Knowledge of the resultant anatomy can guide the surgeon on the management of potential problems. It is relatively rare that patients will know any anatomic details of their surgical procedure, such as whether an alimentary (Roux) limb was placed in an antecolic or retrocolic position. It is therefore useful to obtain any operative reports relevant to the patient’s previous bariatric operation if possible.

Acute care surgeons diagnosing surgical emergencies in postbariatric operation patients must not overlook the common causes of an acute surgical abdomen—acute appendicitis, acute diverticulitis, acute pancreatitis, and gallstone disease—for these are still among the most common etiologies of abdominal pathology in bariatric operation patients. In cases of appendicitis and diverticulitis, a prior bariatric operation may have little impact on the treatment plans or clinical course. Conversely, treatment of pancreatitis and gallstone disease may be significantly impacted by a patient’s resultant anatomy from a bariatric operation, limiting available modalities.

## Early complications

Bariatric procedures are generally safe and effective, but can be associated with devastating complications, some of which may be fatal if not addressed quickly. Bariatric surgical procedures include sleeve gastrectomies (SG), Roux-en-Y gastric bypasses (RYGB), and gastric balloons. Early complications include leaks, stenoses, bleeding, and venous thromboembolic events (VTE). These principles also apply to less commonly performed bariatric operations such as the mini-gastric bypass, single anastomosis duodenal ileal bypass, and the duodenal switch (DS), also known as the biliopancreatic diversion with an SG.

### Leaks

An anastomotic leak is the most dreaded complication of any bariatric procedure because it increases overall morbidity to 61% and mortality to 15%.^1 2^ Failures of anastomotic integrity prolong hospital stays and can result in gastroenteric and gastrobronchial fistulae, which may take months to resolve. Patients undergoing revisional bariatric operations, those who have a body mass index (BMI) of >50 kg/m^2^, and those with dysmetabolic syndrome X are most at risk for leaks.^3–5^ A leak should be suspected and investigated in any patient with persistent tachycardia (>120 beats per minute (bpm)), dyspnea, fever, and abdominal pain. The average time for symptoms of a leak to present is approximately 3 days after the operation.^6^ Often these patients have been discharged home and may present to the emergency room. Sustained heart rates over 120 bpm are a particularly worrisome sign and should be addressed quickly.

Postoperative patients who present with tachycardia and hypotension should be appropriately resuscitated and evaluated for myocardial infarction and pulmonary embolism (PE). Emergency operative exploration should follow if those are ruled out. The operation may be done laparoscopically or open depending on the surgeon’s experience and the severity of the hemodynamic instability. The priorities in the operating room are threefold: removal of contamination, placing closed suction drains to control the leak, and establishment of feeding access. If feasible, closing the leak may be attempted, but it is not required. If a repair is undertaken, interrupted sutures and a modified Graham patch may protect the repair.

In hemodynamically normal patients, evaluation for other causes of postoperative tachycardia, such as postoperative bleeding, hypovolemia, and pneumonia, should precede re-exploration. The evaluation of a leak should include an abdominal CT study with oral contrast; patients should be instructed to drink about 100 cc of contrast just prior to the scan. A CT scan can evaluate for other diseases on the differential diagnosis of the tachycardia, including bleeding and pneumonia. The scan can be performed along with a CT pulmonary angiogram to look for a PE. The detection rate for leaks at the gastrojejunal anastomosis (GJA) or in an SG by CT is 60% to 80%.^6 7^ CT evidence of an abscess, phlegmon, or fluid collection should be considered a leak even if no extravasation of contrast is seen. An upper gastrointestinal series (UGS) can also be used to detect leaks but is less sensitive for a leak at the GJA than a CT,^8^ and neither study will effectively rule out a leak at the jejuno-jejunal anastomosis (JJA) after an RYGB. Persistent tachycardia despite negative radiologic studies warrants surgical exploration if no other cause can be identified due to the poor sensitivities of diagnostic tests. In hemodynamically normal patients, control of a leak may also be done by image-guided drainage.

There are significant differences, however, between the SG leak and the RYGB leak based on the typical endoluminal pressure. After RYGB, the gastric pouch is a low-pressure system, and thus the incidence of leaks ranges from about 0.6% to 4.4% of patients.^9^ Because of this low pressure, operative or non-operative management strategies that control the leak but do not close or repair the perforation are effective in 72% of patients.^10^ Patients who have leaks that last longer than 30 days can be treated with an endoluminal procedure to place clips, stents, or a vacuum dressing to help close these chronic leaks.^11^ Nutrition can be addressed with enteral feeding distal to the GJA and is preferable to total parenteral nutrition. A feeding tube can be placed in the Roux limb, the biliopancreatic limb, or the common channel.

Sleeve leaks, on the other hand, occur in a high-pressure system, are thought to be more common, and range in incidence from 1% to 7%.^12–14^ They are more difficult to treat. Most SG leaks occur at the uppermost extent of the sleeve, where blood supply is tenuous. The high pressure comes from the pyloric and lower esophageal sphincters, or possibly due to a stenosis, twist in the SG, or kink. These anatomic narrowings must be addressed if the leak is to be treated successfully.

Stable patients with leaks after an SG can undergo image-guided drainage procedures. Endoluminal intervention with covered stenting may be placed earlier in the treatment course to help control the leak. The stent should cover from the lower esophageal sphincter (LES) through the pyloric sphincter to allow the leak to heal.^13^ Unfortunately, the most commonly available stents are not long enough (30 cm) to cover this distance.

### Stenosis, twists, or kinks

The loss of luminal caliber from stenosis causes patients to report the sensation of stuck food and the urge to regurgitate. These symptoms are like esophageal dysphagia, with inability to pass food or liquid beyond the GJA or sleeve, and can result in protein calorie malnutrition and nutrient deficiencies. Clinicians must address this when caring for patients with a stenosis, regardless of the cause. Thiamine deficiency can present with new-onset neurologic symptoms. All postsurgical bariatric patients presenting acutely with per os (PO) intolerance should have a neurologic examination, biochemical testing for malnutrition, and nutrition replacement started empirically via an intravenous route because a new neurologic defect can become permanent if not addressed quickly.^15^

RYGB stenosis is common, easy to diagnose, and treatable without another operation. The incidence of stenosis after RYGB is 8% to 19% and is more common after anastomoses done with an end-to-end anastomosis stapler. Comparatively, linear stapled or handsewn anastomoses have fewer strictures.^16^ A UGS will confirm stenosis, showing a failure of contrast to pass through the GJA. Typical management is endoscopic balloon dilation, which can safely be done by an experienced endoscopist within the first week after surgery. The target diameter of the GJA anastomosis after an RYGB is 15 mm in diameter, so patients will have some restrictions when they eat. Anastomoses that are 9 mm or less are stenotic. Serial dilations should be endeavored to achieve optimal size. The diameter should not be increased more than 3 to 4 mm with each treatment, and endoscopists should expect that the dilated diameter will decrease with time. Consequently, most patients will need two to three dilations until they can eat comfortably.^17^

Stenosis after an SG differs from RYGB stenosis in frequency, diagnosis, and therapy. After an SG, true stenosis or stricture occurs infrequently, befalling only 0.69% to 2% of patients.^18–20^ The therapy for a focal stenosis is the same as RYGB stenosis with serial balloon dilations; typically two to three treatments are needed prior to achieving the desired diameter.^20^ Rarely, there is an extensive length of stenosis, which would benefit from 6 weeks of stenting. If this fails to maintain the diameter, a myotomy, either endoscopic or laparoscopic, is the next treatment option.^21^

However, “stenosis” or dysphagia symptoms may develop as a result of a kink in the SG or a volvulus around the SG’s longitudinal axis. Collectively these may occur in up to 9% of patients.^22^ Patients present unable to tolerate PO intake, but the UGS may be completely normal and may not always capture the sleeve in a twist or kink morphology. Additionally, an upper endoscopy may also be normal and allow passage of a 10 mm endoscope because the scope or insufflation air straightens out the twist or kink. Endoscopic interventions will not treat a kink or a volvulus. In these patients, conversion to an RYGB may be the best option, although there are a few reports of using repeat balloon dilation to give the patient a chance to avoid another surgery.^18^ Some SG obstructions are associated with a leak, and as such may impact the timing of operative management. It would be difficult, for example, to perform a conversion RYGB in the operative field full of inflammatory tissue. One may have to stent for 6 weeks to control the leak before attempting a conversion.

### Bleeding

Postoperative bleeding that requires intervention occurs in up to 11% of cases in both the RYGB and SG.^23^ Fortunately, 85% of patients are likely to stop without surgical intervention.^24^ Patients with dysmetabolic syndrome X have a higher risk for bleeding. Usual supportive treatment should be instituted promptly and includes establishing adequate venous access, crystalloid resuscitation, blood product transfusions, serial hematocrits, hemodynamic monitoring, correction of any coagulopathies, and stoppage of VTE chemoprophylaxis if it is being used. An experienced endoscopist can safely evaluate an anastomosis in the early postoperative period and perform therapeutic endoluminal interventions like clips or epinephrine injections as first-line treatment.

Hemodynamic instability or failure of non-operative management mandates emergency surgical management. The staple line is the most common site of bleeding after an SG, but splenic injury is also possible. After RYGB, the anastomoses are probable sites of bleeding, but intra-abdominal hemorrhage from the omentum, mesentery, and spleen are also potential areas. If no obvious site is found, the surgeon must evaluate inside the gastric remnant, the biliopancreatic limb, and the Roux limb for bleeding sources.

### Venous thromboembolism

The rate of a VTE after bariatric operation is low, but a PE is still the most common cause of mortality after these procedures.^25^ Most occur 3 weeks after the procedure,^25^ but there is no indication or consensus about the optimal duration of chemoprophylaxis prescription. There is debate over the risk to these patients, but there is consensus on who the highest risk patients for VTE are: those undergoing revision bariatric operation or open procedures, those with a BMI >50 kg/m^2^, those with surgery duration >4 hours, those with hypercoagulable states, and those with obesity hypoventilation syndrome.^25–27^ When postoperative bariatric patients present acutely in distress, a PE should always be in the differential diagnosis. Screening can be done with a CT angiogram. Treatment consists of systemic anticoagulation, and if a massive embolus is found then a catheter-directed lytic therapy is likely the best treatment option.^28^

### Balloon complications

Acute care surgery providers should probably be familiar with the management of acute complications of balloons used for weight loss. Balloon placements account for less than 1% of bariatric procedures. They are placed endoscopically in the stomach and restrict food intake. They are meant to stay for 6 months or less. Patients will frequently report symptoms of reflux, nausea, and abdominal discomfort even when the balloon is in proper position. About 4% to 7% of patients request early removal because they cannot tolerate these symptoms.^29 30^

Enteric perforation and migration of the balloon leading to a bowel obstruction are two complications which may require acute management and may result in death. Information is sparse, but there does not appear to be anything unique about the presentation of balloon patients with a perforation or bowel obstruction. Deflating a balloon for removal is normally done endoscopically with specialized equipment to puncture the balloon, aspirate the saline, and deflate the balloon. In the instance of migration, the balloon is likely deflated already, but even in the deflated state these balloons are large and may require a sizeable enterotomy to remove them from the intestines. Of note, balloons are inflated with blue-dyed saline, so patients could note blue or green urine if the balloon spontaneously deflates and the blue dye is absorbed from the gastrointestinal tract.^31^ Balloons left in place longer than 6 months are at a higher risk for perforation.^32^

Perforations usually result from pressure necrosis and ulceration from the balloon, and treatment starts with deflating the balloon. In an unstable patient, any large bore needle can be used to deflate the balloon, but a gastrotomy may be needed to gain access to the balloon. The balloon can be decompressed with a large bore endoscopic needle and a snare to extract the balloon. This may cause the dyed saline to spill, making visualization difficult. After the balloon(s) is deflated and removed, the perforation must still be addressed, which can be done with a Graham patch or resection.

## Late complications

### Adjustable gastric band complications

Most band complications are related to mechanical problems with the band itself (eg, band slippage and band, balloon, or tubing breakage). Other and more serious late complications include band erosion, acute obstruction, ischemia, and megaesophagus or pseudoachalasia. Including patients who require band removal for insufficient weight loss, the cumulative incidence of patients requiring reoperation is almost 25%.^33^

#### Band slippage

Band slippage occurs when one wall or side of the stomach slips through the orifice of the band, resulting in a larger than normal gastric pouch superior to the band. The usual anatomic derangement is characterized as ‘cephalad prolapse of the body of the stomach or caudal movement of the band.’^34^ Slippage is considered the most common complication after laparoscopic adjustable gastric band^35^ and occurs in 8% of patients.^36^ Although fundoplication around the band and the pars flaccida technique for placement of the band are thought to reduce the likelihood of band slippage,^37^ it may still occur even after these technical precautions are done at the time of band placement.^38^ Band slippage presents as a dilated gastric pouch superior to the band. These patients often report symptoms of immediate or delayed vomiting after meals, a feeling of fullness only relieved by vomiting, and occasional pain or irritation in the upper abdomen.

**Figure 1:**
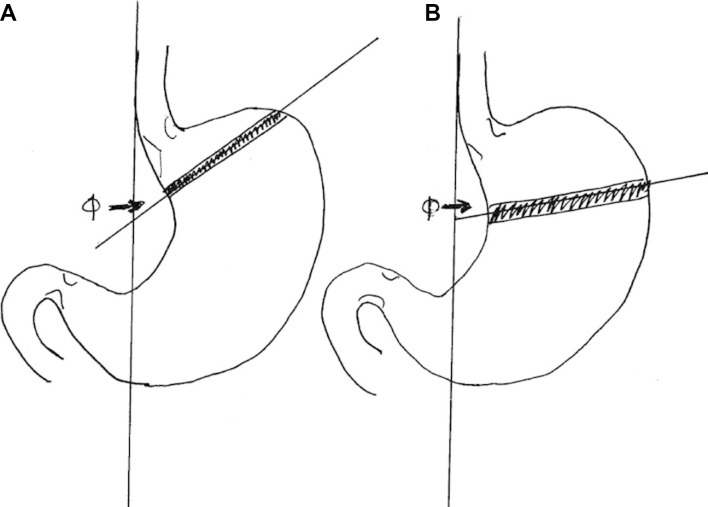
Lap band phi angle (ɸ). (A) Angle of 45° indicating good position. (B) Angle greater than 58° indicating slipped band.

Workup should include a plain abdominal X-ray. The expected band position is to the left of the spinal column with an oblique angle of approximately 15°. This is from 8 o’clock to 2 o’clock when scanning the X-ray from the patient’s right to the left. The “phi angle,” the angle between the vertical spinal column and the band, is normally between 45° and 58° (figure 1). Phi angles greater than 58° usually indicate a slipped band. Seeing the entire ring of band on a plain anterior-posterior abdominal X-ray (the “O sign”)^39^ should also raise suspicion for a slipped band. Additional radiographic signs sensitive for band slippage are inferior displacement of the superior lateral band margin more than 2.4 cm from the diaphragm and the presence of an air-fluid level above the gastric band.^40^

In more severe cases of band slippage, the excess stomach wall herniated through the band orifice may result in swelling and obstruction at the band outlet, resulting in severe dilation and ischemia of the stomach wall above the band. This is like a strangulated hernia. These patients often are completely obstructed and have severe, unrelenting pain, tachycardia, fever, and leukocytosis.

The first treatment step when dealing with a patient with a suspected band complication is to completely empty the band of fluid. In many circumstances, this intervention may resolve the slippage and relieve symptoms. Resolution of band slippage (return of the stomach to its normal position) can be confirmed with a follow-up UGS. Patients who experience relief of symptoms and resolution of band slippage with emptying of the band should be temporarily restricted to a liquid diet and referred to a bariatric surgeon for elective retrieval. Patients who continue to have abdominal pain, systemic signs, or in whom follow-up contrast UGS reveals the band remaining in a slipped position will likely require emergency surgery for band removal and possibly resection of ischemic or necrotic stomach.

Laparoscopic band removal can be challenging. The surgeon will often encounter extensive adhesions of the left lobe of the liver to the upper third of the stomach and a band which appears completely engulfed in stomach tissue. The surgeon’s only indication of the presence of a band may be the band tubing coursing into this area. Careful, persistent dissection allows the left lobe of the liver to be mobilized off the upper stomach and usually is accomplished easily. The next step is identification of the band buckle, which can generally be found on the medial or lesser curvature side of the stomach. Since the band tubing enters near the buckle, following the band tubing will lead to the buckle. Dissection on the buckle itself is necessary to get the band mobile, as there is usually ingrowth of scar tissue in and around the buckle. The silastic balloon portion of the band itself usually resists extensive adhesion formation and will be relatively mobile and easy to slide around the stomach once the buckle is free. Because the buckle is not typically covered with the gastric plication, it is also the area of dissection that is least likely to result in a gastric wall injury.

Once the gastric band is free of adhesions and can be freely rotated around the stomach, it may simply be cut with scissors and removed. The cut band can usually be extracted either through a 15 mm port or via dilation of a smaller port. The tubing and subcutaneous port should also be entirely removed. Prior to completing the exploration, inspection of the posterior gastric wall for ischemia or perforation may identify the need for additional procedures. Plications do not necessarily need to be taken down in the acute setting, although doing so may help assess stomach tissue integrity and potential need for resection. Takedown of the plication in the setting of normal gastric tissue can be safely done either with careful sharp dissection or the use of a linear stapler, with the anvil or narrow side of the stapler placed in the “tunnel” created by the fundoplication and the cartridge side outside the tunnel. The operation is completed with removal of the band’s port in the subcutaneous tissue of the abdominal wall.

#### Band erosion

Although band erosion sounds like an ominous complication, it is rarely a surgical emergency. Erosions occur in a relatively small percentage of patients, ranging from 0.31% to 1.96%.^41 42^ Symptom onset is frequently insidious, vague, and non-specific. Patients may describe upper abdominal or back pain, loss of food restriction, melena, new onset of reflux, or “spontaneous” infection of the subcutaneous band port (from bacteria from the gastric erosion tracking along the band tubing to the subcutaneous port). Plain abdominal X-rays can sometimes document band malposition, and CT scan or upper intestinal contrast series may suggest an intraluminal band and inflammatory changes in the upper stomach. Because the process is slow, adhesion formation around the site of erosion usually limits contamination of the abdomen or peritonitis. Upper endoscopy may document partial or complete erosion of the band into the stomach. When such patients present without sepsis, which is typically the case, they may be started on antibiotics and referred to a bariatric surgeon for management.

Options for treatment depend on the degree of erosion. Complete or near-complete intraluminal bands can be removed endoscopically by cutting the tubing and extracting the band from the mouth.^43 44^ The resultant erosion almost invariably seals quickly due to the slow nature of the erosion and the amount of inflammation present. Similarly, patients with partial erosion may have laparoscopic removal of the band as described above. If a hole is visible, patching with omentum or fundus is usually sufficient to seal it. If a hole is not visible, closed suction drainage, intravenous antibiotics, and a period of nothing by mouth is usually sufficient to seal the erosion. Follow-up UGS can confirm no leak prior to resuming oral intake.

#### Megaesophagus or pseudoachalasia

Megaesophagus or pseudoachalasia rarely requires acute treatment. Patients typically present with worsening dysphagia, regurgitation, or vomiting. Plain X-rays often show the band in normal position, but UGS reveals an esophagus dilated beyond the outer limit of the band. The dilation is attributed to chronic overeating despite having a band to limit intake. As the esophagus expands and the capacity increases, patients describe loss of restriction, which may prompt augmenting the band fill. Additional fill worsens the outlet obstruction and increases the chronic stretching of the esophagus. Initial evaluation and treatment for patients presenting acutely should consist of plain films and UGS to document the problem. Treatment is emptying of the band. These patients should undergo elective band removal.

### Gastric bypass

RYGB results in permanent alteration of anatomy, which provides both the potential for unique complications and can confound the usual treatment options. After ruling out common causes of non-bariatric operation-related complications (appendicitis, diverticulitis and so on), the top four conditions to consider are gallstone disease, marginal ulceration, internal hernia, and intussusception.

#### Gallstone disease

Patients who have had bariatric operation develop gallstones at a higher incidence than the average population.^45^ Alterations in enterohepatic circulation, hormonal changes associated with weight loss, and perhaps increased biliary stasis contribute to the development of cholelithiasis. RYGB results in rerouting of food through the alimentary limb and may change or delay the release of the usual gut hormones that stimulate gallbladder contraction, resulting in atypical symptoms or non-postprandial pain. Symptomatic cholelithiasis and cholecystitis can be treated with laparoscopic cholecystectomy. However, the management of choledocholithiasis is complicated because the usual route to the ampulla of Vater for endoscopic retrograde cholangiopancreatography (ERCP) is bypassed. Pediatric colonoscopes or double-balloon endoscopy can allow highly skilled endoscopists to pass a scope all the way down the alimentary limb through the JJA and back up the biliopancreatic limb to the ampulla of Vater, but this is time-consuming and not always in the armamentarium of the endoscopist.

Hence, the three options available to the surgeon for treatment of choledocholithiasis after gastric bypass are percutaneous transhepatic cholangiography, surgical common bile duct exploration, or the so-called “rendezvous” procedure where the surgeon laparoscopically provides access to the bypassed stomach remnant to allow the gastroenterologist to approach the ampulla of Vater with a standard side-viewing ERCP scope. Biliopancreatic diversion/DS patients have only the first two options, as these patients typically do not have retained stomach for this access. Some institutions have created algorithms to treat these patients that require complex multidisciplinary procedures.^46^

#### Marginal ulceration

Just under 5% of patients develop marginal ulceration after RYGB.^47^ It typically occurs at or near the GJA, although typical peptic ulcers in the first portion of the duodenum have also been described.^48^ The most frequent symptoms are epigastric burning pain occurring in approximately 57% of patients, followed by bleeding in 15%.^47^

#### Perforation

Patients may present with spontaneous perforations (1%–2% of patients). Some may have no warning symptoms, although a detailed history may reveal antecedent symptoms of postprandial pain and nausea or recent increased use of either non-steroidal anti-inflammatory drugs (NSAIDs) or tobacco. Risk factors for perforation include smoking, NSAID use, and anastomosis with non-absorbable suture material.^49^

In the setting of acute perforation in a patient with a remote history of bariatric operation, the diagnosis is often suspected based on the history and physical examination alone. Patients who have fever, tachycardia, and peritonitis on examination may need no additional workup (or at most a plain abdominal X-ray demonstrating free air) before committing them to operating exploration. Patients may be managed laparoscopically or open; the priorities are to reduce contamination and control the leak. Omental patch repair of the defect is acceptable with or without primary closure of the perforation and closed suction drainage. In this setting major revision operations should be avoided, if possible.

Patients with less clear-cut presentations may require abdominal CT. Like hemodynamically stable patients with early leaks, localized or contained perforations in patients without sepsis and intact immune systems can be managed non-operatively with intravenous antibiotics, proton pump inhibitors, bowel rest, and careful observation for the development of sepsis. Like early leaks from the GJA or gastric pouch staple line, late marginal ulcer perforations can also be managed with endoscopic placement of intraluminal stents and percutaneous and image-guided drainage of accessible intra-abdominal fluid collections in selected patients.

#### Bleeding

Mild to moderate bleeding from marginal ulcers occurs in 5% of patients; massive hemorrhage is substantially less common.^50^ Presentation is like any patient with upper gastrointestinal bleeding and includes melena or hematochezia, hematemesis, and near-syncope or syncope. Initial management should focus on resuscitation with crystalloid or blood products if appropriate, reversal of antiplatelet agents or anticoagulants, and intravenous proton pump inhibitors. Upper endoscopy is diagnostic and usually therapeutic. Bleeding is commonly identified at the GJA site, and the majority can be controlled with standard endoscopic techniques. In one study, surgery was only required in 4% of patients with bleeding marginal ulcer.^51^ Since most patients who require operative management have pathology not amenable to endoscopic therapy, surgical treatment should consist of resection of the ulcer site (usually the GJA) with revision of the anastomosis in healthy tissue. Combined laparoscopic and endoscopic procedures, where an endoscopically identified isolated bleeding vessel is laparoscopically oversewn without opening the lumen, have been successfully performed.

After control of the hemorrhage, patients should be counseled that strict abstinence from smoking and NSAIDs is mandatory to minimize the chance of recurrence. Patients with non-healing ulcers or large/dilated gastric pouches may need to be referred to a bariatric surgeon for elective revision operation.

#### Small bowel obstruction

RYGB patients may develop small bowel obstructions related to internal hernias or postoperative adhesions. More rarely, stenosis of the JJA, small bowel bezoars, and small bowel intussusception (often at the jejuno-jejunostomy site) may lead to obstructions in these patients. Classic presentation is with diffuse abdominal pain, distension, bloating, nausea, and vomiting. Vomiting may be less pronounced than non-gastric bypass patients. Bowel obstruction related to adhesions is more common after open procedures. In patients who have had a prior laparoscopic gastric bypass, over 50% of small bowel obstructions are caused by internal hernias.^52^

#### Internal hernia

Perhaps the most difficult to identify but potentially catastrophic late complication in post-RYGB patients is an internal hernia with small bowel volvulus. Symptoms may be non-specific and intermittent. Axial imaging may be read as negative or normal in about 30% of patients.^53^ Vital signs and laboratory values may be relatively normal unless vascular compromise of intestinal tissue has already occurred.

**Figure 2:**
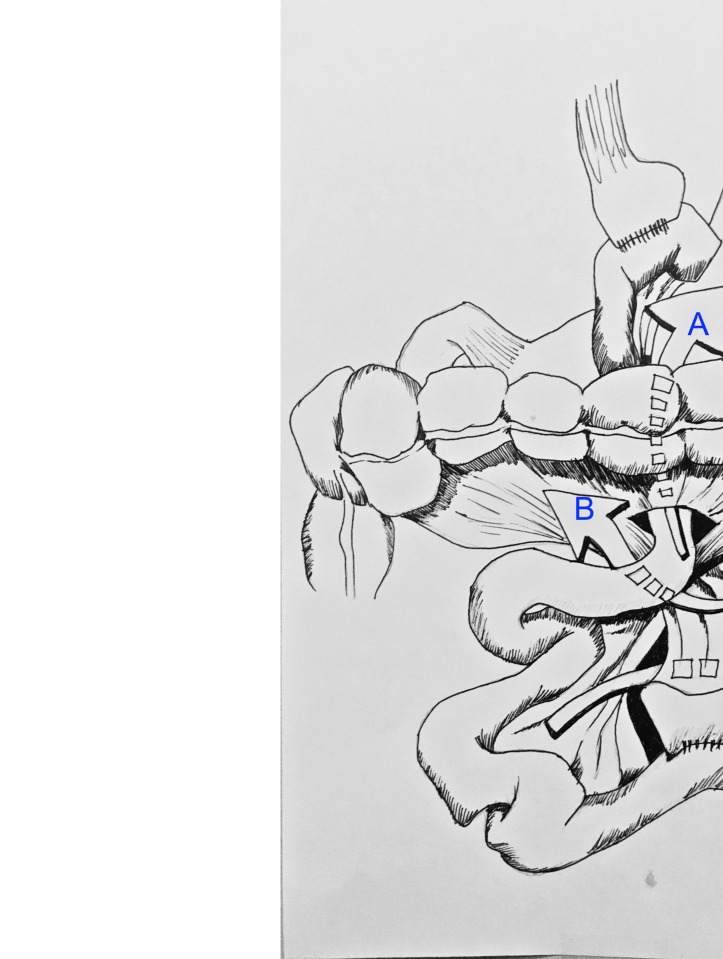
Internal hernias of retrocolic Roux-en-Y gastric bypass. (A) Transverse mesocolic defect. (B) Petersen’s defect. (C) Jejuno-jejunostomy mesenteric defect.

Internal hernias after bariatric operation can occur at anastomotic sites, but can also occur through the transverse mesocolic defect in the setting of a retrocolic alimentary or Roux limb arrangement. The defect that occurs between the alimentary (Roux) limb mesentery and the transverse mesocolon is known as the Petersen’s defect (figure 2). There is also a defect at the mesentery of the JJA. Closure of these defects at the time of initial operation is thought to reduce their incidence, but even with prophylactic closure, internal herniation and volvulus can still occur. The overall incidence of internal hernias after RYGB is 2.5%, with the majority (87%) of hernias occurring at either the transverse mesocolic defect or Petersen’s defect.^54^

Patients with internal hernia and small bowel volvulus typically present with mid-epigastric or periumbilical abdominal pain, often of relatively sudden onset. Their pain may be unremitting and radiate to the back. Eating can often worsen symptoms, and in advanced cases symptoms of a bowel obstruction with obstipation and vomiting may be reported. Symptoms may be general enough that providers evaluating the patients may consider marginal ulcers or symptomatic gallstones in their differential diagnoses, leading to evaluations with upper endoscopy or abdominal ultrasounds and potentially delaying therapy.^55 56^ Cross-sectional imaging may reveal telltale signs of internal hernia, such as mesenteric swirl or obstructive patterns and engorged mesenteric nodes.^57^ However, CT imaging has a suboptimal sensitivity for internal hernias in patients with a history of bariatric operation and may be read as normal in up to 30% of patients.^54^

Post-RYGB patients in whom small bowel obstructive symptoms are present, or in whom imaging reveals a small bowel obstruction, should generally not have a trial of non-operative management. Blind nasogastric tube placement can easily result in perforation of the blind end of the alimentary limb, but typically will not correct a bowel obstruction related to an internal hernia. These patients should be taken expeditiously to the operating room.^58^

As with all postbariatric operation problems, knowledge of the patient’s operative anatomy prior to exploration is helpful (eg, antecolic vs. retrocolic alimentary limb). Patients with small bowel volvulus through an internal hernia defect will often have what appears to be a “knot” or twist of bowel loops in the infracolic abdomen, and it can be difficult to ascertain which direction to run the bowel to get it reduced. This conundrum can be addressed by starting at the terminal ileum and running the bowel retrograde. This will usually both reduce the volvulus and allow clear delineation of the problem. If all the bowel is viable, simple closure of the internal defect should suffice. Surgeons should inspect all possible mesenteric defects for adequate closure. Typically, about 70% of internal hernias can be corrected laparoscopically, but surgeons should not hesitate to convert to open operation if laparoscopic reduction and repair of an internal hernia is not progressing safely. Devitalized bowel should be resected.

## Conclusion

Acute care surgeons can safely care for bariatric patients, including many of the complications related to their weight loss procedure. The threshold to operate to in these patients, in general, should be lower when they present with acute symptoms but not without understanding the specific circumstances.
